# Daily sleep duration and risk of metabolic syndrome among middle-aged and older Chinese adults: cross-sectional evidence from the Dongfeng–Tongji cohort study

**DOI:** 10.1186/s12889-015-1521-z

**Published:** 2015-02-24

**Authors:** Jing Wu, Guiqiang Xu, Lijun Shen, Yanmei Zhang, Lulu Song, Siyi Yang, Handong Yang, Yuan Liang, Tangchun Wu, Youjie Wang

**Affiliations:** MOE Key Lab of Environment and Health, School of Public Health, Tongji Medical College, Huazhong University of Science & Technology, Hangkong Road 13, Wuhan, 430030 Hubei China; Department of Maternal and Child Health, School of Public Health, Tongji Medical College, Huazhong University of Science & Technology, Hangkong Road 13, Wuhan, 430030 Hubei China; Dongfeng General Hospital, Dongfeng Motor Corporation and Hubei University of Medicine, Daling Road 16, Shiyan, 442000 Hubei China; Department of Social Medicine, School of Public Health, Tongji Medical College, Huazhong University of Science & Technology, Hangkong Road 13, Wuhan, 430030 Hubei China

**Keywords:** Metabolic syndrome, Daily sleep duration, Daytime napping duration, Nighttime sleep duration

## Abstract

**Background:**

Evidence from epidemiological studies has demonstrated that a shorter or longer duration of nighttime sleep may increase the risk of metabolic syndrome. Little is known about the association between daily sleep duration, including nighttime sleep and daytime napping duration, and metabolic syndrome. We aimed to examine the association between daily sleep duration and metabolic syndrome and its components in middle-aged and older Chinese adults using data from the Dongfeng-Tongji Cohort study.

**Methods:**

A total of 25,184 participants (mean age 63.6 years) who completed the baseline questionnaire, physical examination and laboratory tests were included in this analysis. Daily sleep duration was calculated by summing up nighttime sleep duration and daytime napping duration. The metabolic syndrome was defined using the International Diabetes Federation criteria. Logistic regression models were used to investigate the association between daily sleep duration and the risk of metabolic syndrome and its components.

**Results:**

Of the participants, 8,046 (31.9%) had metabolic syndrome. Females had a higher prevalence (38.6%) of metabolic syndrome than males (23.9%). Female participants with longer daily sleep duration (≥8 hours, all *P* < 0.05) per day had a higher risk of metabolic syndrome compared with those sleeping 7–7.9 hours, adjusting for potential confounders. Longer daily sleep was positively associated with individual components of metabolic syndrome except central obesity in females, and was only positively associated with HDL-C in males. Further analysis revealed that a longer duration of daytime napping (≥90 minutes, *P* < 0.05) was associated with the risk of metabolic syndrome in females. However, nighttime sleep duration was not associated with the risk of metabolic syndrome in either males or females.

**Conclusions:**

Our findings suggested that longer daytime napping duration rather than nighttime sleeping duration was associated with increased risk of metabolic syndrome in females. The findings have significant implications for further studies to explore the appropriate sleep duration for middle-aged and older adults.

## Background

Metabolic syndrome (MetS), a clustering of physiologically related cardiovascular risk factors, including central adiposity, dyslipidemia, elevated blood pressure and increased glucose level, is a critical public health problem worldwide [1]. The syndrome is closely correlated with the development of chronic kidney disease, cardiovascular disease, and a higher risk of all-cause mortality [2-4]. In China, the age-standardized prevalence of MetS was 9.8% in males and 17.8% in females [5]. Given high prevalence of MetS, it is significant to identify modifiable risk factors to prevent MetS.

Sleep is a basic need for human, and closely related to the body’s metabolism. Previous studies have indicated that short or long sleep duration is associated with an increased risk for MetS, but the findings are not consistent [6]. Studies have demonstrated that older adults had different sleep pattern compared with younger adults [7,8]. A meta-analysis performed by Flyd et al. found that sleep duration increased with age while sleep disturbances also increased [7], suggesting that older adults may have more daily sleep duration throughout the day. However, to date, there is little evidence about the effects of daily sleep duration on human health outcomes for older adults.

Daytime napping is traditionally regarded as a healthy life behavior and is especially good for older adults in China. The percentage of older Chinese adults who practice habitual daytime napping reaches 61.7% in men and 46.8% in women [9]. Accumulative evidence showed that daytime napping might be a marker of underlying health risks. A large cohort in British revealed that excessive daytime napping was associated with higher risk of mortality from all-cause and respiratory diseases [10]. Xu *et al.* demonstrated an independent association between longer durations of daytime sleep and the risk of diabetes in older adults [11]. Given the high prevalence of daytime napping for older adults in China, it is of great importance to examine the effects of daytime napping for health outcomes.

Evidence from studies have found different effects of daytime napping and nighttime sleep for health [11,12], therefore, in this study, we aimed to examine the associations between daily sleep duration, including daytime napping and nighttime sleep with MetS in a Chinese middle-aged and older population based on the data from the Dongfeng-Tongji cohort study of retired workers.

## Methods

### Study participants

Data for our analysis was derived from the Dongfeng-Tongji Cohort study (DFTJ cohort) which was initiated in 2008 among retired employees of Dongfeng Motor Corporation (DMC) in Shiyan City, Hubei Province. The rationale, design, and methods of the DFTJ cohort have previously been described in detail [13]. Between 2008 and 2010, 87% (27009/31000) of the invited retired employees responded to the study and completed baseline information, medical examinations and provided blood samples. For current analysis, the participants who had missing data on sleep habits or the determination of the status of MetS were excluded. Thus, the final sample included 25184 subjects. The demographic characteristics including sex and age were similar between individuals included in the current study and those excluded. This study was approved by the Medical Ethics Committee of the School of Public Health, Tongji Medical College, Huazhong University of Science and Technology and Dongfeng General Hospital, DMC. All participants signed an informed consent form.

### Assessment of MetS

We classified participants as having MetS if they have central adiposity (defined as waist circumference ≥ 90 cm for Chinese males and ≥ 80 cm for Chinese females) plus any two of the following four factors according to the new International Diabetes Federation (IDF) definition [1]: a. triglycerides (TG) ≥1.7 mmol/L or specific treatment for this lipid abnormality; b. high-density lipoprotein cholesterol (HDL-C) <1.03 mmol/L in males or < 1.29 mmol/L in females or specific treatment for this lipid abnormality; c. systolic blood pressure (SBP) ≥ 130 mmHg or diastolic blood pressure (DBP) ≥ 85 mm Hg or treatment of previously diagnosed hypertension; d. fasting plasma glucose (FPG) ≥ 5.6 mmol/L or previously diagnosed type 2 diabetes.

### Assessment of nighttime sleep, daytime napping and daily sleep

Nighttime sleep duration was assessed by asking: “On average, how many hours and minutes do you sleep per night?”. Nighttime sleep duration was classified as <7 hours, 7–7.9 hours, 8–8.9 hours, and ≥9 hours. Habitual daytime nappers were defined as those who had taken a planned or regular nap as a habit more than three times per week after lunch over the past twelve months. Daytime napping was assessed by asking “Do you have a habit of taking a nap after lunch?” Those who reported “Yes” were further asked about the average duration of their naps. Daytime napping duration was categorized as no daytime napping, <30 minutes, 30–59 minutes, 60–89 minutes, and ≥90 minutes. Additionally, we computed daily sleep duration by summing up nighttime sleep duration and daytime napping duration, and we categorized it into five categories: <7 hours, 7–7.9 hours, 8–8.9 hours, and 9–9.9 hours and ≥10 hours.

### Assessment of covariates

Demographic information on age, sex, education level (elementary or below, junior high school, high school, college or above), and marital status (e.g., married, unmarried, widowed, divorced) was included in the questionnaire. History of physician-diagnosed chronic diseases and history of medication were also obtained. The criteria for family history of diabetes or hypertension were having at least one first-degree relative with a diagnosis of diabetes or hypertension. Health-related habits on smoking status, alcohol drinking status, and physical activity was also obtained. Physical activity was defined as those who exercise more than 20 min per day and more than three times per week over the last six months. The anthropometric and laboratory data included weight, height, waist circumference, systolic and diastolic blood pressure, high density lipoprotein, and triglyceride. Body mass index was calculated as weight in kilograms divided by height in meters squared.

### Statistical analysis

Numerical variables were summarized as mean ± SD and categorical data were presented as proportion (%). Student *t*-tests were used to examine difference in means for the numerical data between participants with MetS and those without MetS, and Chi-square tests were used for categorical data. According to several studies, the prevalence of MetS displayed a gender difference that females have a higher prevalence of MetS than males [5,14]. Moreover, females reported to have more sleep disturbances than males in older adults [15]. Therefore, we conducted a gender-stratified analysis. Logistic regression analyses were used to estimate the odds ratios (ORs) and 95% confidence intervals (95% CIs) of daily sleep duration with MetS in males and females, respectively. We used participants with daily sleep duration of 7–7.9 hours as the reference group. Model 1 was adjusted for demographic factors (age, marriage, education), heath-related life behaviors (smoking status, drinking status, physical activity), self-reported physician-diagnosed chronic diseases and a family history of diabetes or hypertension. Model 2 was adjusted for the variables in Model 1 plus BMI. Moreover, to better understand and interpret the results of the association between daily sleep duration groups and MetS, we further explored the effects of daytime napping duration or nighttime sleep duration on MetS by performing logistic regression analyses adjusting for covariates similar to the above-mentioned models. We used participants with nighttime sleep duration of 7–7.9 hours and those who did not nap in the daytime as references respectively in examining the associations among nighttime sleep duration and daytime napping duration with MetS. In addition, multivariable logistic regression was also used to test the relationship between daily sleep duration and the components of MetS in males and females, respectively. All statistical analysis was performed using SPSS software (version 11.0). Associations were considered statistically significant at *P <* 0.05.

## Results

Of the 25,184 participants (11,370 males, 13,814 females), 8,046 (31.9%) had MetS, females had a higher prevalence (38.6%) of metabolic syndrome than males (23.9%). Participants with MetS were more likely to be non-smokers, nondrinkers, and had less physical activity (Table 1). A higher proportion of participants with MetS had physician diagnosed chronic diseases (all *P <* 0.001 for listed diseases) compare with those without MetS. In addition, participants with MetS were more likely to have higher blood pressure, higher level of triglycerides, and higher body mass index.

**Table 1:** **The characteristics of study participants with and without the metabolic syndrome (n = 25,184)**

	**Participants with MetS**	**Participants without MetS**	
**Characteristics**	**(n = 17,138)**	**(n = 8,046)**	***P***
Age (year)	64.6 ± 7.7	63.2 ± 7.8	<0.001
Sex			<0.001
Male (%)	2720 (33.8)	8650 (50.5)	
Female (%)	5326 (66.2)	8488 (49.5)	
Marital status			<0.001
Married (%)	7025 (87.5)	15518 (90.8)	
Widowed (%)	845 (10.5)	1172 (6.9)	
Unmarried (%)	22 (0.3)	54 (0.3)	
Divorced (%)	133 (1.7)	350 (2.0)	
Education			<0.001
Elementary or below (%)	2789 (34.9)	4598 (27.1)	
Junior high school (%)	2922 (36.6)	6058 (35.6)	
High school (%)	1641 (20.6)	4362 (25.7)	
College or above (%)	630 (7.9)	1979 (11.6)	
Physical activity (%)	6454 (80.2)	14067 (82.1)	<0.001
Current smoker (%)	1011 (12.7)	3496 (20.5)	<0.001
Current drinker (%)	1275 (15.9)	3997 (23.3)	<0.001
Self-reported medical history			
Coronary heart disease (%)	1775 (22.3)	2121 (12.5)	<0.001
Myocardial infarction (%)	278 (3.5)	423 (2.5)	<0.001
Stroke (%)	443 (5.6)	605 (3.6)	<0.001
Hypertension (%)	4623 (57.5)	5023 (52.1)	<0.001
Diabetes mellitus (%)	1712 (21.3)	1383 (8.1)	<0.001
Family history of diabetes (%)	470 (6.0)	64.4 (5.1)	0.004
Family history of hypertension (%)	2008 (25.3)	3498 (20.8)	<0.001
Body mass index (kg/m^2^)	27.0 ± 3.1	23.3 ± 2.9	<0.001
Waist circumference (cm)	91.0 ± 7.5	79.3 ± 7.9	<0.001
Fasting plasma glucose (mmol/L)	6.6 ± 2.1	5.8 ± 1.5	<0.001
Systolic blood pressure (mmHg)	136.1 ± 18.0	126.6 ± 18.1	<0.001
Diastolic blood pressure (mmHg)	80.8 ± 11.1	76.3 ± 10.5	<0.001
HDL-C (mmol/L)	1.3 ± 0.4	1.5 ± 0.4	<0.001
Triglycerides (mmol/L)	5.3 ± 1.0	5.1 ± 0.9	<0.001

Data are presented as mean ± SD or number (percentage).

Abbreviations: *MetS* metabolic syndrome, *HDL-C* high-density lipoprotein cholesterol.

Table 2 presents the prevalence of MetS in each sleep duration category by gender. Using Chi-square test, we found a higher prevalence of MetS in participants with longer duration of daily sleeping than those with daily sleep duration of 7–7.9 hours in females. There was statistically significance between different nighttime sleep groups and the prevalence of MetS in females. Moreover, female participants with longer duration of daytime napping had a higher prevalence of MetS compared with those who did not nap. Daily sleep duration, nighttime sleep duration and daytime duration had no effects on the prevalence of MetS in males.

**Table 2:** **Prevalence of metabolic syndrome by sleep duration stratified by gender**

	**Male**		***P***	**Female**		***P***
	**Non-MetS**	**MetS**		**Non-MetS**	**MetS**	
**Daily sleep duration (hours)**			0.12			<0.001
<7	236 (72.6)	89 (27.4)		370 (63.8)	210 (36.2)	
7-7.9	1048 (76.4)	324 (23.6)		1473 (66.3)	749 (33.7)	
8-8.9	2652 (76.4)	818 (23.6)		2928 (62.5)	1759 (37.5)	
9-9.9	2772 (77.0)	827 (23.0)		2380 (59.6)	1613 (40.4)	
≥10	1942 (74.6)	662 (25.4)		1337 (57.3)	995 (42.7)	
**Nighttime sleep duration (hours)**			0.19			0.005
<7	599 (73.0)	222 (27.0)		738 (64.3)	410 (35.7)	
7-7.9	2346 (76.3)	727 (23.7)		2470 (63.1)	1445 (36.9)	
8-8.9	3510 (76.4)	1083 (23.6)		3529 (60.4)	2316 (39.6)	
≥9	2195 (76.1)	688 (23.9)		1751 (60.3)	1155 (39.7)	
**Daytime napping duration (minutes)**			0.08			<0.001
None	2426 (77.6)	700 (22.4)		2996 (63.9)	1690 (27.9)	
<30	218 (74.9)	73 (25.1)		372 (72.1)	144 (27.9)	
30-59	1281 (76.8)	386 (23.2)		1515 (17.8)	922 (37.8)	
60-89	2625 (75.6)	849 (24.4)		2303 (27.1)	1577 (40.6)	
≥90	2100 (74.7)	712 (25.3)		1302 (15.3)	993 (43.3)	

Abbreviations: *MetS* metabolic syndrome.

The associations between duration of daily sleep and Mets stratified by genders were displayed in Table 3. Model 1 showed that females participants with a daily sleep duration of 8–8.9 hours (OR, 1.16 [95% CI, 1.04-1.30]), 9–9.9 hours (OR, 1.22 [95% CI, 1.08-1.36]), and ≥10 hours (OR, 1.29 [95% CI, 1.13-1.46]) had a significantly higher odds ratio (OR) for MetS compared with females with daily sleep duration of 7–7.9 hours after adjustment for demographic factors (age, marriage, education), heath-related life behaviors (smoking status, drinking status, physical activity), self-reported physician-diagnosed chronic diseases and a family history of diabetes or hypertension. In model 2, after adjusting for BMI which is a main known risk factor for MetS, the ORs were 1.20 (95% CI, 1.06-1.37), 1.28 (95% CI, 1.12-1.46), and 1.33 (95% CI, 1.15-1.54) for females who slept 8–8.9 hours, 9–9.9 hours, ≥10 hours, respectively, compared with females who slept 7–7.9 hours. To better understand and provide valuable clues to explain the relationship between daily sleep duration and MetS, we further analyzed nighttime sleep duration or daytime napping duration on the risk of MetS using a series of logistic regression models (Table 3). Nighttime sleep duration was not associated with MetS in all three models after adjusting for demographic factors, heath-related life behaviors, disease history, and daytime napping duration in either males or females. With regard to the association between daytime napping duration and MetS, model 1 showed that longer daytime napping durations of ≥ 90 minutes (OR, 1.22 [95% CI, 1.09-1.36]) had a risk of MetS compared with those who never napped in the daytime in females, while shorter napping time (<90 minutes) had no effects on the risk of MetS (Table 3), after adjusting for demographic factors, heath-related life behaviors, self-reported physician-diagnosed chronic diseases and a family history of diabetes or hypertension. Model 2 showed that longer napping duration of ≥ 90 minutes (OR, 1.17 [95% CI, 1.08-1.27]) still was associated with higher risk of MeSt after adjustment for BMI in females. When nighttime sleep duration was added in model 3, the risk of MetS was moderately attenuated (OR, 1.20 [95% CI, 1.06-1.37]). Daily sleep duration, nighttime sleep duration and daytime sleep duration were not associated with MetS among males.

**Table 3:** **Adjusted odds ratios (95% CI) for metabolic syndrome by sleep duration stratified by gender**

	**Model 1**	**Model 2**	**Model 3**
**Daily sleep duration (hours)**			
**Male**			
<7	1.21 (0.91-1.61)	1.28 (0.90-1.80)	
7-7.9	1.00	1.00	
8-8.9	0.99 (0.85-1.16)	1.14 (0.95-1.37)	
9-9.9	0.94 (0.80-1.09)	1.12 (0.93-1.34)	
≥10	1.08 (0.92-1.26)	1.10 (0.98-1.38)	
**Female**			
<7	1.13 (0.92-1.39)	1.06 (0.84-1.35)	
7-7.9	1.00	1.00	
8-8.9	1.16 (1.04-1.30)	1.20 (1.06-1.37)	
9-9.9	1.22 (1.08-1.36)	1.28 (1.12-1.46)	
≥10	1.29 (1.13-1.46)	1.33 (1.15-1.54)	
**Nighttime sleep duration (hours)**			
**Male**			
<7	1.22 (1.01-1.46)	1.15 (0.93-1.44)	1.04 (0.93-1.17)
7-7.9	1.00	1.00	1.00
8-8.9	0.99 (0.89-1.11)	1.13 (0.99-1.29)	1.04 (0.97-1.12)
≥9	1.01 (0.89-1.14)	1.28 (1.10-1.49)	1.01 (0.94-1.10)
**Female**			
<7	0.96 (0.83-1.11)	0.94 (0.79-1.11)	0.93 (0.79-1.10)
7-7.9	1.00	1.00	1.00
8-8.9	1.08 (0.99-1.18)	1.06 (0.96-1.17)	1.05 (0.96-1.16)
≥9	1.04 (0.93-1.15)	1.10 (0.97-1.24)	1.10 (0.97-1.24)
**Daytime napping duration (minutes)**			
**Male**			
None	1.00	1.00	1.00
<30	1.05 (0.79-1.40)	0.94 (0.67-1.33)	0.94 (0.67-1.33)
30-59	1.03 (0.89-1.19)	1.07 (0.90-1.28)	1.07 (0.90-1.28)
60-89	1.09 (0.97-1.23)	1.05 (0.91-1.22)	1.05 (0.91-1.22)
≥90	1.12 (1.00-1.28)	1.09 (0.94-1.27)	1.09 (0.94-1.27)
**Female**			
None	1.00	1.00	1.00
<30	0.92 (0.73-1.10)	0.87 (0.75-1.02)	0.88 (0.74-1.03)
30-59	1.02 (0.92-1.14)	1.03 (0.97-1.20)	1.03 (0.91-1.16)
60-89	1.10 (1.00-1.21)	1.08 (0.97-1.20)	1.08 (0.97-1.20)
≥90	1.22 (1.09-1.36)	1.21 (1.06-1.37)	1.20 (1.06-1.37)

Model 1, adjusted for age, marriage, education, smoking status, drinking status, physical activity, coronary heart disease, myocardial infarction, stroke, and family history of hypertension or diabetes.

Model 2, adjusted for the variables in model 1 plus body mass index (BMI).

Model 3, adjusted for the variables in model 2 plus daytime napping duration or nighttime sleep duration, as appropriate.

The logistic regression analyses of the relationship between daily sleep duration and the components of MetS showed that long daily sleep duration was independently associated with its components as well except for central obesity in females. Among males, longer daily sleep duration was associated with higher risk of HDL-C criteria only (Table 4).

**Table 4:** **Adjusted odds ratios of the components of metabolic syndrome by daily sleep duration per day stratified by gender**

	**Duration of daily sleep (hours/day)**				
	**<7**	**7~**	**8~**	**9~**	**≥10**
**Male**					
Central obesity criterion	1.26 (0.95-1.65)	1.00	0.93 (0.80-1.07)	0.90 (0.80-1.05)	0.89 (0.77-1.04)
Glucose criterion	0.93 (0.71-1.22)	1.00	0.92 (0.81-1.06)	0.99 (0.87-1.15)	1.10 (0.96-1.28)
Blood pressure criterion	0.87 (0.65-1.16)	1.00	0.98 (0.84-1.13)	0.95 (0.82-1.10)	1.05 (0.90-1.67)
HDL-C criterion	1.26 (0.88-1.80)	1.00	1.23 (1.01-1.49)	1.22 (1.01-1.48)	1.40 (1.15-1.71)
Triglycerides criterion	1.00 (0.75-1.35)	1.00	0.99 (0.85-1.15)	1.12 (0.97-1.30)	1.17 (0.99-1.36)
**Female**					
Central obesity criterion	1.05 (0.85-1.29)	1.00	1.11 (0.99-1.24)	1.08 (0.96-1.21)	1.04 (0.91-1.19)
Glucose criterion	0.81 (0.66-1.00)	1.00	0.98 (0.88-1.09)	0.96 (0.86-1.08)	1.18 (1.04-1.34)
Blood pressure criterion	1.00 (0.80-1.24)	1.00	1.08 (0.96-1.22)	1.10 (0.97-1.24)	1.18 (1.02-1.36)
HDL-C criterion	0.99 (0.79-1.24)	1.00	1.02 (0.90-1.15)	1.14 (1.01-1.29)	1.21 (1.05-1.39)
Triglycerides criterion	1.07 (0.85-1.33)	1.00	1.05 (0.93-1.18)	1.11 (0.98-1.26)	1.15 (1.00-1.32)

Adjust for age, marriage, education, smoking status, drinking status, physical activity, coronary heart disease, myocardial infarction, stroke, and the other four components of metabolic syndrome as appropriate.

HDL-C, high-density lipoprotein cholesterol.

## Discussion

With a large sample size, we provided epidemiological evidence that long daily sleep duration (≥8 hours) is associated with MetS, while short daily sleep duration (<7 hours) was not associated with MetS in middle-aged and older Chinese females after adjustment for potential confounders. Moreover, longer daily sleep duration appears to have an adverse effect on the components of the MetS, including triglyceride, HDL-C, blood pressure and glucose. Interestingly, further analysis revealed that longer daytime napping (≥90 minutes) rather than longer nighttime sleep is independently associated with a higher risk for MetS.

Few studies have investigated the relationship between the combined sleep duration of nighttime sleep and daytime napping and MetS. The findings of the current study were consistent with one recent study which reported that Korean females who slept ≥9 h/d had 1.43 times higher risk of MetS compared with those who slept 7 h/d [16]. Teresa *et al.* explored the relationship between total sleep duration and MetS in 29,333 Chinese adults ages 50–96 years and found that participants who slept longer (≥8 h/d) were more likely to have MetS and its components [17]. However, Teresa *et al.* did not revealed gender-specific associations between total sleep duration and MetS or further analyze the relationships among nighttime sleep duration, daytime napping duration and MetS. The reasons for the gender-specific associations are unclear. One explanation may be hormonal differences and the effects of hormones on sleep [18]. Middle-age females progressing the menopausal transition is significantly associated with difficulty sleeping [19]. Older females with decreasing estrogen levels were found to be associated with more trouble falling asleep and frequent awakening [15]. Thus, the association between long sleep duration and MetS may be amplified in females. In addition to the physiological explanation, there have been reported that differential social-economic characteristics and social roles of males and females could lead to the difference of sleep quality [20].

Our findings indicated that neither long nor short nighttime sleep is related to the prevalence of MetS in females and males. The association between nighttime sleep duration and MetS is controversial. Some studies indicated that both short and long sleep duration is correlated to the risk of MetS, suggesting a U-shaped pattern [21,22]. Several studies demonstrated that the risk of MetS increased with short nighttime sleep duration only [23-25]. Another study reported that only long nighttime sleep duration was associated with elevated risk for MetS [26]. There were several possible reasons for observing a nonsignificant relationship between nighttime sleep duration and MetS in our study. First, unlike the studies mentioned above, all the participants of our study were middle-aged and older retired adults who have more time to sleep and less chance for sleep restriction due to retirement. Only 7.8% of participants slept less than 7 hours per night and only 3.7% of participants slept less than 6 hours per night, which may have contributed to the lack of power in this study. Second, as for the components of MetS, we found that longer nighttime sleep duration was a risk factor for reduced HDL-cholesterol and elevated triglycerides, while it was a protective factor for central obesity (data not shown). This opposing direction of the associations between nighttime sleep duration and the components of MetS might be one explanation for the absence of a relationship between nighttime sleep duration and MetS.

Few studies have examined the association between daytime napping and MetS. Lin *et al.* found that a longer daytime napping duration but not a longer nighttime sleeping duration was associated with a higher risk for MetS in females, which was consistent with our findings [12]. Compared with the study of Lin *et al.*, our study has the strength of large sample size and abundant covariates. Data from 16,480 older adults from the Guangzhou Biobank Cohort Study revealed that a higher frequency of daytime napping was independently related to an increased risk of MetS [11]. However, they assessed the frequency of daytime napping rather than the duration of it. The mechanism underlying the association between longer daytime napping duration and MetS is unclear. Sleep could have various influences on metabolism and endocrine secretion patterns by interfering with the circadian rhythm [27,28]. Circadian rhythm disturbances arising from excessive daytime napping could be one explanation for the relationship between longer daytime napping duration and MetS. Circadian rhythm disturbances can lead to insulin resistance, which plays a major role in the development of MetS and is almost always present with other metabolic abnormalities [29,30]. Another physiologic pathway which plays a role in the relationship between daytime napping and MetS may be sympathetic activation [31]. It has been reported that the practice of napping can induce the elevation of blood pressure mediated by sympathetic activation [32]. Indeed, the sympathetic nervous system is crucial in circulatory and metabolic control and was found to be associated with MetS [33]. Previous studies have reported that long daytime sleep duration was associated with an increased risk for cardiovascular mortality in the elderly [10,34]. The presence of MetS carries an elevated risk of cardiovascular disease [3]. Therefore, our results may provide a mechanism linking longer daytime sleep duration and cardiovascular mortality via mediation of MetS.

Several potential limitations of our study need to be mentioned. First, we collected the information about sleep duration based on self-reports. Despite high correlations between subjective estimates of sleep duration and more direct assessments such as actigraphy [35,36], other objective measurements of sleep duration are needed to support our findings. Secondly, the cross-sectional design limits causal inferences on the association between sleep duration and MetS. However, in the Chinese context, daytime napping is considered healthy and most Chinese develop the habit of daytime napping in youth [37], making the reverse association unlikely in this case. Third, we did not include other potential confounders that could affect sleep patterns such as obstructive sleep apnea (OSA). OSA is independently correlated with insulin resistance and a high risk of MetS, and is known to cause excessive sleepiness [38,39], with habitual nappers having a higher frequency of OSA [40]. Missing data on these crucial factors could inevitably lead to potential bias in the evaluation of the odds ratios for MetS. Finally, this study was performed in a Chinese population, and our results might not be able to be generalized to other races and ethnic groups.

The strengths of our study include a large sample size; the availability of blood measurements; the abundant data on potential confounding factors; and standardized questionnaires, which not only enhance the precision but also allow statistical adjustment for multiple variables.

## Conclusions

Longer daytime napping duration rather than nighttime sleep duration is associated with a moderate elevated risk of MetS in females. The findings have significant implications for further study to examine the appropriate duration of sleep for older adults especially for females.
